# Midwifery

**Published:** 1837-01

**Authors:** 


### MIDWIFERY.

### *On Binding the Abdomen of Lying-in Women.* By Hugh Ley, M.D.

Dr. Ley is of opinion that the value of the bandage so commonly applied after delivery has been greatly over-rated. In his experience there is but one circumstance in which the bandage with a compress over the uterus is imperatively called for. It is in a form of hemorrhage described by Dr. Gooch, in which the uterus which had contracted is again dilated by hemorrhage taking place into its cavity: in these cases, it is not only requisite that the uterus should be cleared of the coagula, but that it should be prevented from again enlarging; and this can only be done by a bandage and compress; but they should not be applied until repeated gushes of blood have reduced the circulation. In preventing the occurrence of hemorrhage, for which it has been advised, its use is very equivocal; for, amongst the poor where it is omitted the relative mortality from hemorrhage is probably less than among the rich. To restrain actual hemorrhage it is obviously insufficient; and, if it may have been applied, it must be removed, in order to ascertain the state of the uterus, and to adopt those manipulations by which the bleeding is effectually restrained.

The bandage alone may be applied over the abdomen, with moderate and equal pressure, in cases of syncope following rapid delivery, which is produced, as in tapping, from the sudden removal of pressure; and, in such circumstances, it should be applied immediately on the expulsion of the child. It may also be used to satisfy ladies who believe that it will preserve their form by preventing permanent distention of the abdomen, and to relieve uneasy sensations consequent on the relaxed condition of the abdominal muscles; but, in these cases, it need not be applied until the clothes have been changed, and the patient placed in bed, and sufficiently loose to allow the hand to pass easily between it and the belly.

The bandage and compresses often produce *injurious consequences.* Dr. Ley has known it force the uterus to one side of the abdomen, producing much uneasiness in the hip, stretching the ligaments and pressing the appendages of the uterus against the sharp linea ileo-pectinea: hence laying the foundation of permanent obliquity of the uterus, not unfrequently a cause of barrenness. Another effect is to push the uterus lower into the cavity of the pelvis; thus producing prolapsus uteri, which Dr. Ley has more than once traced to this cause. Not only is the uterus pushed downwards, but the vagina is sometimes thrown into folds; the whole circumference descending until it forms a bulky tumour at the vulva. These evils are great, and not infrequent. Dr. Ley is also inclined to think that the bandage increases after-pains.

*Med. Gazette, September* 10 and 17, 1836.

---

### *On the Spontaneous Amputation of the Limbs of the Fœtus in Utero.* By J. G. Simpson, M.D.

This curious subject was investigated by Dr. Montgomery in the first and second volumes of the Dublin Journal, and illustrated by two cases which had come under his own observation. From these Dr. M. concluded, that this spontaneous amputation is the consequence of the constriction of the limb at the point of separation, by a ligature of organized lymph. In the present paper, Dr. Simpson adduces a good many additional instances of the same circumstance from authors, and notices also three cases which had recently come under his own observation. Dr. S. adopts the explanation of this curious phenomenon advanced by Dr. Montgomery, and adduces some additional arguments in its support. The following are Dr. S.'s conjectures as to the manner in which the partial or total separation of the limb is affected. He assumes with Dr. Montgomery, that the effusion of the lymph in the first instance is the result of inflammation, although how or wherefore this is produced he cannot explain. It is necessary to recollect, (says Dr. S.,) how readily the atrophy or interstitial absorption of any living texture is produced by the application to it of a continuous and strong pressure, and that, in the earlier months, the limbs of the fœtus are so slender, and their component tissues so soft. Now, supposing the organizable lymph to be once effused, so as to have its two extremities attached to two parts of the body, more or less distant from one another—as to two parts of two limbs,—it is evident that the texture of the pseudo-membrane must soon become stretched, and compress more or less precisely those parts over or around which it passes, in proportion as the two points of the body forming its origin and insertion become gradually more and more separated from one another, in the regular progress of development. The sudden movements of the fœtus may also contribute to the same effect. *Dublin Journal, November,* 1836.

